# Lenacapavir and the unfinished work of HIV prevention literacy in Nigeria

**DOI:** 10.4102/phcfm.v18i1.5687

**Published:** 2026-09-16

**Authors:** Oluwafemi P. Owodunni

**Affiliations:** 1 Department of Emergency Medicine, School of Medicine, The University of New Mexico, Albuquerque, United States of America

Nigeria introduced semi-annually injectable lenacapavir for pre-exposure prophylaxis (PrEP) in March 2026, with initial implementation in eight states.^1^ This rollout strategy enters a prevention landscape marked by concentrated human immunodeficiency virus (HIV) risk and limited PrEP awareness. Despite prior implementation efforts, only 36.1% of adolescents and young people from key populations were aware of oral PrEP; in a separate survey of Nigerian youth, 62.6% have never heard of PrEP.^2,3^ Furthermore, programmatic surveillance also found that 84.0% of oral PrEP users discontinued within 30 days.^4^ These findings highlight the limits of regimen convenience alone; although lenacapavir may reduce daily pill burden, its population impact will depend on sustained public education and implementation strategies that support uptake and medication adherence.

## Investigation and outcomes

Google Trends is a publicly available, anonymised data source and was queried using web search categories from 01 January 2026 to 26 June 2026. The platform reports sampled activity as normalised relative search volume (rSV), scaled from 0 to 100 for a given geography and period. It does not represent absolute search counts, and equivalent values across geographical locations may reflect different search volumes.^5^ Therefore, rSV is interpreted as an ecological measure of online information seeking rather than a direct measure or validated predictor of lenacapavir uptake. Across Africa, higher lenacapavir rSV was concentrated in countries with early rollout activity. South Africa had the highest relative interest, followed by Kenya, Zambia, and Eswatini. Nigeria, Uganda, Mozambique, Zimbabwe, and Lesotho showed moderate activity, whereas most other countries had low or insufficient measurable interest (Figure 1). Within Nigeria, search activity remained low and episodic, with brief increases around rollout. These peaks may reflect media coverage or implementation activity rather than demand (Figure 1).

**FIGURE 1:**
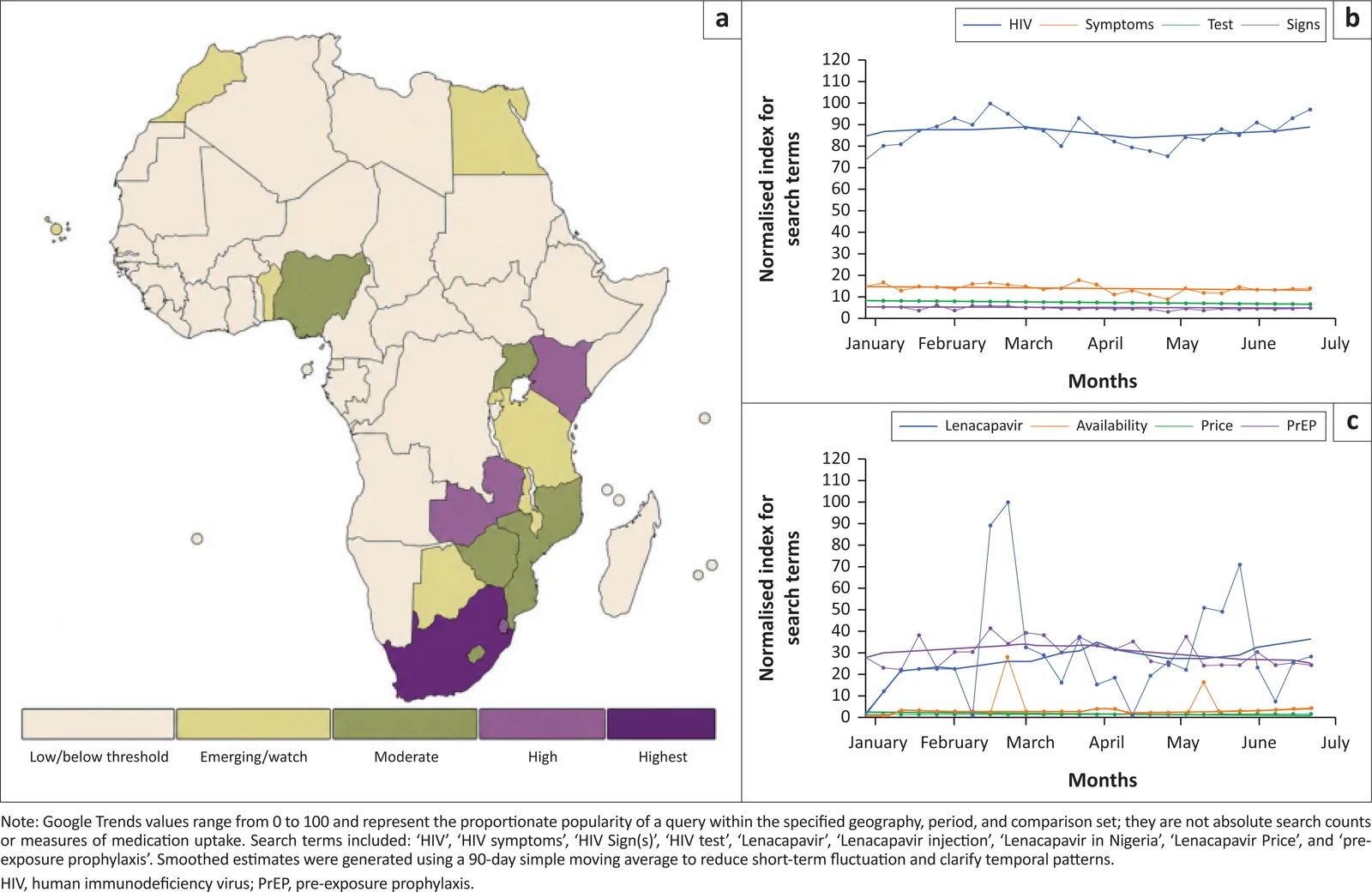
Geographic and temporal patterns in Google search interest for lenacapavir and human immunodeficiency virus prevention during early rollout in Nigeria compared to countries across Africa from January 2026 to June 2026. (a) Presents country-level normalised relative search volume for ‘Lenacapavir’ across Africa (colour intensity reflects hierarchical categories). (b & c) Present Nigeria-specific search activity for the terms listed in each panel.

## Preliminary conclusions and actions

Google Trends data suggest that digital visibility remained limited and inconsistent during early implementation. These findings cannot determine initiation, geographic access, stock continuity, or uptake among priority populations. However, scaling lenacapavir within a setting of low PrEP awareness and limited counselling capacity may constrain its reach. Effective rollout will require clinician readiness to prescribe, clear referral and access pathways, reliable supply, and multilingual, stigma-responsive education through digital and print media, pharmacies, maternal-child health services, and peer networks. Search monitoring may identify information gaps and assess communication efforts; however, the barriers that have limited oral PrEP uptake may similarly constrain the population-level impact of lenacapavir unless identified and addressed early in implementation.
